# The Role of AaPGRP-LB in the Immune Response of *Aedes albopictus* Against Bacteria Infection

**DOI:** 10.3390/ijms26052188

**Published:** 2025-02-28

**Authors:** Cheng Wu, Yahui Chen, Chenhua Zheng, Xitong Huang, Yuyang Xie, Lingqun Lin, Xiuli Zhang, Lihua Xie

**Affiliations:** 1 School of Basic Medical Sciences, Fujian Medical University, No. 1 Xuefu North Road, Fuzhou 350122, China; wucheng@fjmu.edu.cn (C.W.); yhchen_1220@163.com (Y.C.); zhengchenhua@fjmu.edu.cn (C.Z.); 13194060772@163.com (X.H.); Xyy20040327@163.com (Y.X.); 18450192880@163.com (L.L.); 2 College of Integrative Medicine, Fujian University of Traditional Chinese Medicine, No. 1 Qiuyang Road, Fuzhou 350122, China

**Keywords:** *Aedes albopictus*, peptidoglycan recognition protein, PGRP-LB, immune response

## Abstract

The initial phase of an insect’s innate immune response to foreign pathogens is triggered by the identification of exogenous invaders, a mechanism facilitated by pattern recognition receptors. Among these receptors, peptidoglycan recognition proteins (PGRPs), abundant in insects, are essential components of the innate immune system. The roles of PGRPs have been extensively elucidated in *Drosophila melanogaster*; however, the mechanism underlying the immune response of *Aedes albopictus* to pathogens is unclear. Herein, we successfully cloned the full-length cDNA of a PGRP gene from *Ae. albopictus*, designated as the *AaPGRP-LB* gene. The open reading frame of *AaPGRP-LB* encodes 203 amino acids, including a secretion signal peptide and a canonical PGRP conserved domain. Multisequence alignment revealed that AaPGRP-LB possesses the amino acid residues essential for zinc binding and amidase activity. Molecular docking studies demonstrated that AaPGRP-LB exhibits a strong binding affinity for DAP-type and LYS-type peptidoglycan. The mRNA expression level of the *AaPGRP-LB* gene significantly increased after oral infection with *Escherichia coli* or *Staphylococcus aureus*. The purified recombinant AaPGRP-LB (rAaPGRP-LB) exhibited strong agglutination properties and demonstrated significant antimicrobial efficacy against *E. coli* and *S. aureus* in the presence of zinc ions. This study highlights the critical role of AaPGRP-LB in the immune response of *Ae. albopictus*. These findings provide a foundation for future research on mosquito immune pathways for innovative vector control and disease prevention strategies.

## 1. Introduction

*Aedes albopictus* (Skuse), classified in the subgenus Stegomyia of the family Culicidae (Diptera), is a highly invasive species that has proliferated across more than 129 countries [1]. This species is a medically important vector for viruses responsible for diseases that pose significant global public health challenges and economic burdens, including dengue, chikungunya, and Zika viruses [2,3]. The ability of *Ae. albopictus* to transmit these pathogens primarily depends on its capacity to identify them as foreign, initiating various immune responses to combat the pathogens. Pathogen recognition is predominantly facilitated by a class of proteins known as pattern recognition receptors, which include a subset of proteins termed peptidoglycan recognition proteins (PGRPs) [4].

PGRPs were initially identified and isolated from the hemolymph of the silkworm, *Bombyx mori*, where they were discovered to activate the antibacterial function of the phenoloxidase proenzyme by specifically recognizing and binding to peptidoglycan in bacterial cell walls [5]. Since this discovery, over 100 members of the PGRP family have been identified across various organisms, including insects and humans [6,7,8,9,10]. PGRPs are highly conserved proteins, each containing at least one conserved PGRP domain, approximately 165 amino acid residues in length at the carboxy terminus. This domain is homologous to N-acetylmuramyl-alanine amidase and exhibits approximately 30% sequence similarity with bacteriophage T7 lysozyme [11,12]. PGRPs can be classified based on molecular weight into short (S) and long (L) types and by the presence or absence of signal peptides. Short-type PGRPs (PGRP-S) usually have a signal peptide and function as secretory proteins, while long-type PGRPs (PGRP-L) generally lack signal peptides but may possess a transmembrane domain, existing as intracellular or membrane-bound proteins [13,14].

PGRPs are essential for recognizing invading pathogens and regulating innate immune responses, and they can directly eliminate intruders. PGRPs can identify peptidoglycans (PGNs), which are differentiated into L-lysine type (LYS)-PGNs and meso-diaminopimelic acid type (DAP)-PGNs based on differences in the third amino acid residue of their peptide chains [15]. Subsequently, the Toll signaling, Imd, and JAK-STAT pathways, reactive oxygen metabolism, and melanization reaction are selectively activated to combat pathogens [16,17,18]. Recent studies have demonstrated that PGRPs play a crucial role in antifungal and antiviral activities [19,20].

In *Drosophila melanogaster*, 13 *PGRP* genes have been identified, comprising 7 *PGRP-S* and 6 *PGRP-L* genes, whose functions in host defense are well-characterized [8,21]. In *D. melanogaster*, DmPGRP-SA and DmPGRP-SD interact with the Gram-positive bacteria binding protein to activate the Toll signaling pathway [22]. The transmembrane receptor DmPGRP-LC in *D. melanogaster* recognizes DAP-PGNs on Gram-negative bacteria and forms a complex with DmPGRP-LE, an intracellular receptor, culminating in the activation of the Imd signaling pathway [23,24]. In *B. mori*, 12 *PGRP* genes have been identified, comprising 6 *PGRP-S* and 6 *PGRP-L* genes [25]. BmPGRP-S2 participates in the IMD pathway, facilitating antimicrobial peptide (AMP) expression [26,27]. BmPGRP-S4 binds to and hydrolyzes both types of PGNs in the presence of Zn^2+^, thereby enhancing the prophenoloxidase cascade [28]. BmPGRP-L1 is associated with AMP expression and functions as a receptor in the midgut, binding to both types of PGNs while lacking amidase activity [14]. *BmPGRP-L4* is significantly upregulated in the midgut, epidermis, fat body, and silk glands after oral infection with *Escherichia coli* and *Staphylococcus aureus* [29]. *Aedes aegypti* possesses 7 *PGRP* genes, including 2 *PGRP-S* and 5 *PGRP-L* genes. *Ae. aegypti* PGRP-LB negatively regulates AMP gene expression, including defensin, cecropin, and gambicin [30]. In *Ostrinia furnacalis*, OfPGRP-A primarily detects Gram-positive bacteria and is potentially involved in the Toll signaling pathways, while OfPGRP-B identifies Gram-negative bacteria and may participate in the Imd signaling pathways [31]. The function of PGRPs in *Ae. albopictus* remains unexplored.

Herein, we amplified the open reading frame (ORF) of the *AaPGRP-LB* gene from *Ae. albopictus* and conducted a comprehensive bioinformatics analysis. We analyzed the mRNA expression levels of *AaPGRP-LB* in response to pathogen challenges, including *E. coli* and *S. aureus*. Furthermore, the recombinant AaPGRP-LB (rAaPGRP-LB) protein was expressed in prokaryotic cells, and its agglutination activity and antibacterial effects against *E. coli* and *S. aureus* were evaluated. These findings provide a new understanding of the role of AaPGRP-LB in the innate immune system of *Ae. albopictus* and may facilitate the development of biopesticides for targeted control of invasive agricultural pests.

## 2. Results

### 2.1. cDNA Cloning and Characterization Analysis of AaPGRP-LB

**Figure 1:**
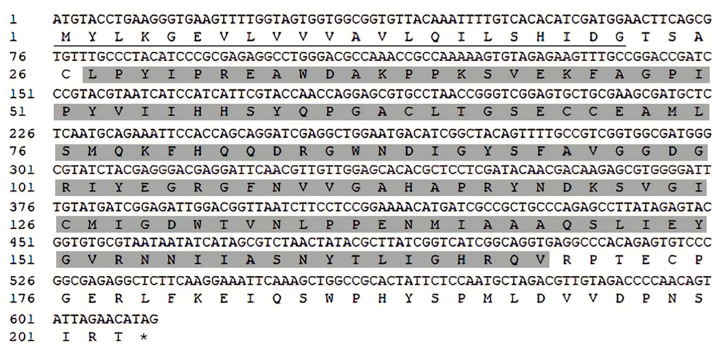
Analysis of the sequence of *Aedes albopictus PGRP-LB* (*AaPGRP-LB*). The nucleotide and corresponding amino acid sequences of *AaPGRP-LB*. The signal peptide is indicated by an underline, while the PGRP conserved domain is highlighted. The numbers in the upper row correspond to the positions in the nucleotide sequence, while those in the lower row represent the positions in the amino acid sequence. The asterisk (*) indicates a stop codon, which is not translated into an amino acid.

The ORF of the *AaPGRP-LB* gene was inferred from compiled RNA-Seq data (NCBI Reference Sequence: XM_019708127.2). Specific primers were designed based on this ORF prediction, and polymerase chain reaction (PCR) amplification was performed for the *AaPGRP-LB* gene. The gene was effectively isolated from *Ae. albopictus* Fuzhou strain, revealing an ORF of 612 nucleotides that encodes a peptide comprising 203 amino acid residues, including a 22-residue signal peptide (Figure 1). The cDNA sequence of *AaPGRP-LB* has been deposited in the GenBank database (GenBank accession number: OR689811.1).

### 2.2. Structure and Phylogeny of AaPGRP-LB

**Figure 2:**
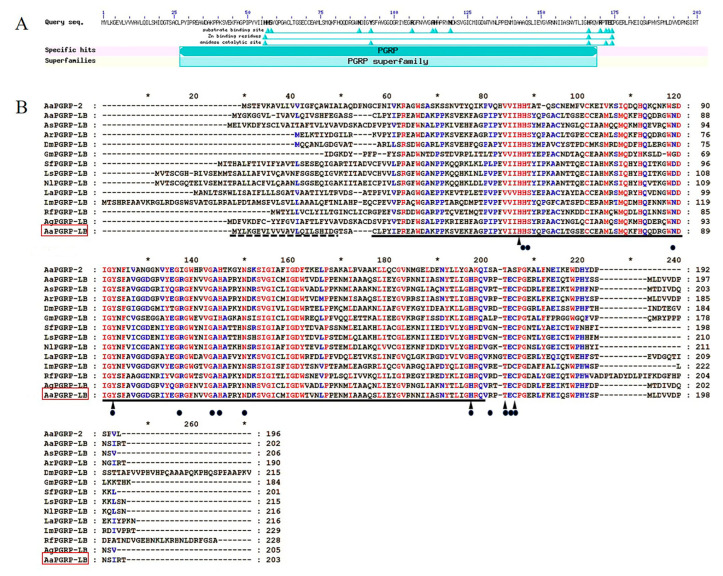
Analysis of the structure of AaPGRP-LB and multiple sequence alignment with other insect PGRP-LBs. (**A**) Conserved Domain Database (CDD) analysis reveals the conserved domain characteristic of PGRP proteins. (**B**) Amino acid sequences of insect PGRP-LBs were obtained from the Genebank: AaPGRP-2, *Aedes aegypti* (XM_001660053.3); AaPGRP-LB, *Ae. aegypti* ((XM_001654225.3); AsPGRP-LB, *Anopheles stephensi* (XM_036047282.1); ArPGRP-LB, *Armigeres subalbatus* (JF828728.1); DmPGRP-LB, *Drosophila melanogaster* (NM_141822.3); GmPGRP-LB, *Galleria mellonella* (AM392402.1); SfPGRP-LB, *Sogatella furcifera* (MW323547.1); LsPGRP-LB, *Laodelphax striatellus* (KU866523.1); NlPGRP-LB, *Nilaparvata lugens* (KC355211.1); LaPGRP-LB, *Lasioderma serricorne* (KU866523.1); LmPGRP-LB, *Locusta migratoria* (KX342009.1); RfPGRP-LB, *Rhynchophorus ferrugineus* (MG457266.1); AgPGRP-LB, *Anopheles gambiae* (XM_061648309.1); and AaPGRP-LB, *Ae. albopictus* (OR689811.1). The red square denotes the target sequence, the dashed line indicates the signal peptide, the solid line denotes the PGRP conserved domain, the black triangle signifies the amidase active site, and the black dots represent conserved amino acid residues responsible for recognizing peptidoglycan. Numbers indicate the positions in the amino acid sequence. Red indicates fully conserved residues, blue denotes conservative substitutions, and black represents non-conservative substitutions.

Studies demonstrate that catalytic PGRPs, homologous to bacteriophage T7 lysozyme and bacterial type 2 amidases, typically possess a conserved set of five amino acid residues, HIS-TYR-HIS-THR-CYS, essential for Zn^2+^ dependent amidase activity [32,33,34]. In *D. melanogaster* PGRP-LB (DmPGRP-LB), an active amidase, interacts with Zn^2+^, with the T residue as a surface amino acid at the active site for PGN binding [35]. Analysis of the AaPGRP-LB using National Center for Biotechnology Information-Conserved Domain Database (NCBI-CDD) and InterPro identified a PGRP superfamily domain at the N-terminal, which includes a substrate binding site (HIS-57, SER-58, ASN-88, TYR-92, ARG-106, ALA-113, HIS-114, ASN-119, HIS-166, ARG-170, THR-172, GLU-173, and CYS-174), zinc-binding residues (HIS-56, HIS-166, and CYS-174), and an amidase catalytic site (HIS-56, TYR-92, HIS-166, THR-172, and CYS-174) (Figure 2A). The deduced protein has an estimated molecular weight of 22.5 kDa, and a theoretical pI of 6.15. Figure 2B illustrates that multiple sequence alignments identified five conserved amino acid residues in AaPGRP-LB, essential for amidase activity, consistent with findings from 13 other insect PGRP-LBs.

**Figure 3:**
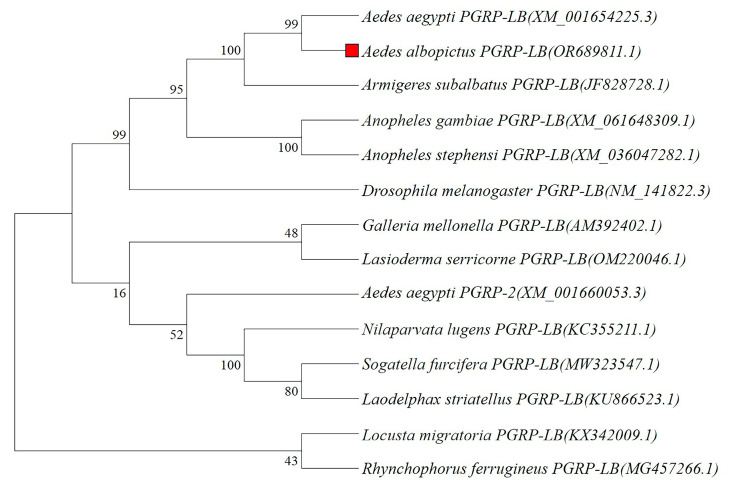
Phylogenetic analysis of AaPGRP-LB with other known insect PGRPs. The following protein sequences were used: *Ae. aegypti* PGRP-LB (XM_001654225.3); *Ae. albopictus* PGRP-LB (OR689811.1); *Ar. subalbatus* PGRP-LB (JF828728.1); *An. gambiae* PGRP-LB (XM_061648309.1); *An. stephensi* PGRP-LB (XM_036047282.1); *D. melanogaster* PGRP-LB (NM_141822.3); *G. mellonella* PGRP-LB (AM392402.1); *L. serricorne* PGRP-LB (KU866523.1); *Ae. aegypti* PGRP-L2 (XM_001660053.3); *N. lugens* PGRP-LB (KC355211.1); *S. furcifera* PGRP-LB (MW323547.1); *L. striatellus* PGRP-LB (KU866523.1); *L. migratoria* PGRP-LB (KX342009.1); and *R. ferrugineus* PGRP-LB (MG457266.1). The numbers on the branches represent the percentage of 1000 bootstrap. The red square represents the target sequence.

A phylogenetic analysis was performed to clarify the evolutionary relationships among PGRPs from different insect species. Figure 3 illustrates that PGRP-LB from *Ae. albopictus* and *Ae. aegypti* clustered together, exhibiting high statistical support (99% bootstrap confidence). Additionally, they constituted a more extensive clade with DmPGRP-LB, corroborated by 99% bootstrap confidence. These findings confirm the classification of AaPGRP-LB within the peptidoglycan recognition protein family and suggest that AaPGRP-LB exhibits amidase activity capable of hydrolyzing bacterial peptidoglycan.

### 2.3. Protein Structure Prediction and Docking with the PGN Ligand

**Figure 4:**
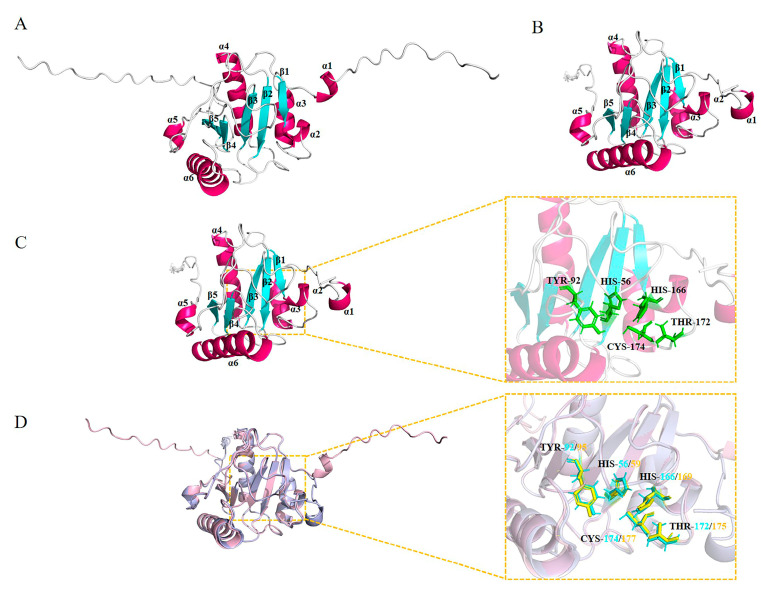
The three-dimensional structures of DmPGRP-LB and AaPGRP-LB. (**A**) DmPGRP-LB. (**B**) AaPGRP-LB. In these structures, α-helices are illustrated in hot pink, and β-strands are depicted in cyan. (**C**) The positions of the five amidase active sites in AaPGRP-LB are marked in green. (**D**) The superimposition of the positions of the five amidase active sites in AaPGRP-LB (green) and DmPGRP-LB (yellow). Numbers indicate the positions in the amino acid sequence.

We utilized AlphaFold (version 2.3.2) to construct three-dimensional models of the structural domains of PGRP for DmPGRP-LB (Figure 4A) and AaPGRP-LB (Figure 4B,C). Over 90% of the amino acid residues in AaPGRP-LB were located in the most favored regions, indicating the successful development of high-quality 3D structures for AaPGRP-LB. The results indicate that the molecule exhibits analogous architectures, each comprising six α-helices (α1, α2, α3, α4, α5, and α6) and five β-strands (β1, β2, β3, β4, and β5). The root mean square deviation derived from the values of the superposition of DmPGRP-LB and AaPGRP-LB was 0.275 (Figure 4D). Furthermore, the catalytic centers of DmPGRP-LB and AaPGRP-LB were aligned and compared, revealing a remarkably similar overall architecture between the two proteins.

**Figure 5:**
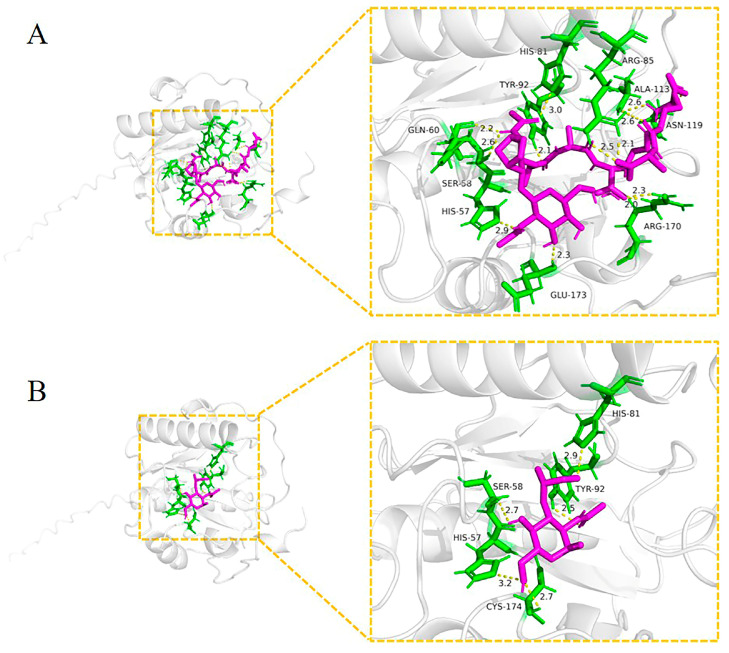
The docking view illustrates AaPGRP-LB binding to various ligands. (**A**) Interaction with TCT (tracheal cytotoxin) and (**B**) Interaction with MTP (muramyl tripeptide). Hydrogen bonds are represented by yellow dashed lines, and ligands are illustrated as magenta sticks. The interacting residues of the receptor are depicted as green sticks. The residues forming hydrogen bonds with the ligands are as follows: (**A**) HIS-57, SER-58, GLN-60, TYR-72, HIS-81, ARG-85, ALA-113, ASN-119, ARG-170, and GLU-173. (**B**) HIS-57, SER-58, HIS-81, TYR-92, and CYS-174. Numbers indicate the positions in the amino acid sequence.

Docking studies were performed using Autodock Vina software (version 1.1.2) to evaluate the ability of AaPGRP-LB to identify PGNs. Two ligands, representing DAP-type PGN (tracheal cytotoxin, TCT) (Figure 5A) and LYS-type PGN (muramyl tripeptide, MTP) (Figure 5B), were utilized to interact with the amino acids of AePGRP-LB. The binding energies for these ligands were determined to be −31,380 and −26,778 J/mol, respectively, indicating strong interactions.

### 2.4. The mRNA Expression Status of the AaPRP-LB Gene After Pathogen Challenge with E. coli and S. aureus

**Figure 6:**
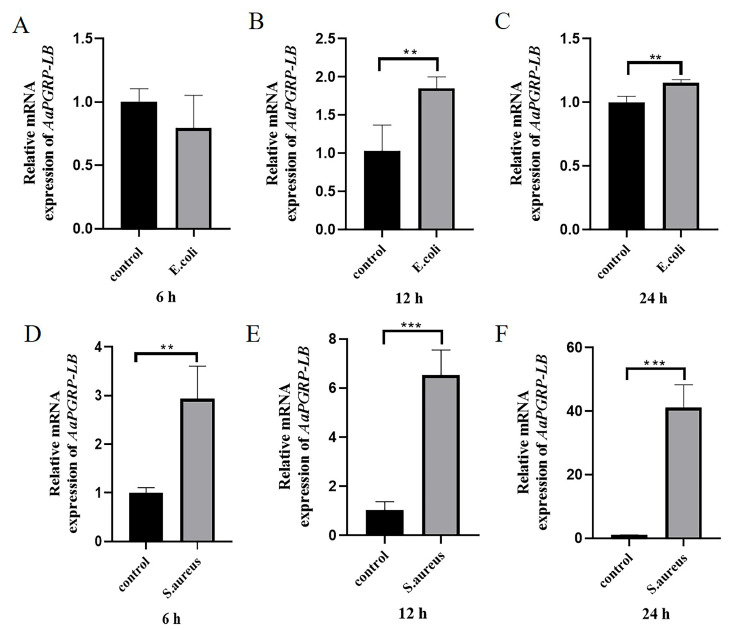
Analysis of the expression levels of *AaPGRP-LB* in *Ae. albopictus* at different timepoints after oral bacterial challenge. (**A**–**C**) Expression levels of *AaPGRP-LB* after oral *Escherichia coli* challenge. (**D**–**F**) *AaPGRP-LB* expression level after oral *Staphylococcus aureus* challenge. The bars represent the mean ± standard deviation (SD) of three independent experiments (n = 3). Asterisks denote a statistically significant difference compared to the control group, as determined by an unpaired *t*-test (** *p* < 0.01; *** *p* < 0.001).

The mRNA expression levels of the *AaPGRP-LB* gene in *Ae. albopictus* were valuated using real-time quantitative polymerase chain reaction (RT-qPCR) after oral infection with *E. coli* and *S. aureus*. The findings demonstrated that *AaPGRP-LB* gene relative expression did not increase at 6 h (*t* = 1.303, *p* > 0.05) (Figure 6A) but was significantly upregulated at 12 h (*t* = −3.868, *p* < 0.01) (Figure 6B) and 24 h (*t* = −4.915, *p* < 0.01) (Figure 6C) following *E. coli* challenge. However, in response to *S. aureus* challenge, the *AaPGRP-LB* gene exhibited a continuous upregulation in expression at 6 h (*t* = −4.895, *p* < 0.01) (Figure 6D), 12 h (*t* = −8.799, *p* < 0.001) (Figure 6E), and 24 h (*t* = −9.703, *p* < 0.001) (Figure 6F). These findings suggest that *AaPGRP-LB* is induced by bacterial infection through the oral route.

### 2.5. Recombinant Expression and Purification of AaPGRP-LB Protein

**Figure 7:**
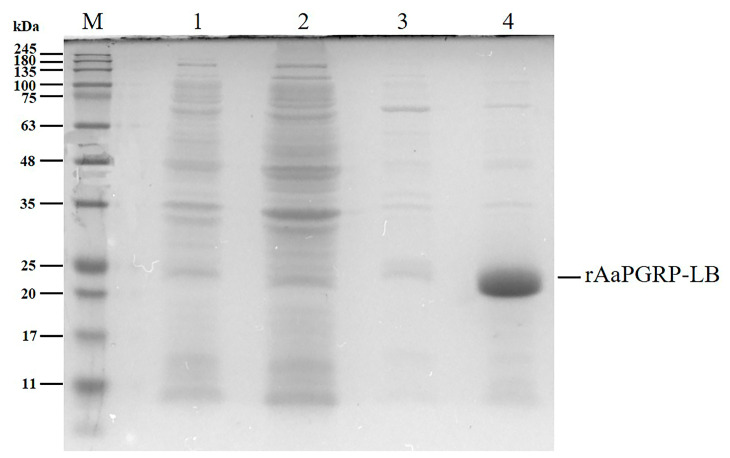
Isopropyl β-D-1-thiogalactopyranoside (IPTG)-induced expression and affinity chromatography purification of recombinant AaPGRP-LB analyzed by SDS-PAGE. M, protein marker (kDa); 1, the control group (without IPTG induction) demonstrates the proteins expressed by the host cells under normal conditions; 2, the flow-through from the unbound Ni-nitrilotriacetic acid (Ni-NTA) resin column, indicating non-specifically bound proteins; 3, the proteins eluted with 20 mM imidazole elution buffer, which likely includes weakly bound contaminants; 4, the protein eluted with 300 mM imidazole elution buffer, representing the purified recombinant AaPGRP-LB.

The expression vector pET28a-His-AaPGRP-LB, containing the cDNA sequence of AaPGRP-LB without the signal peptide-encoding segment, was successfully constructed and introduced into *E. coli* BL21(DE3). The recombinant His-tagged protein was induced using isopropyl β-D-1-thiogalactopyranoside (IPTG), followed by purification through affinity chromatography with a nickel-nitrilotriacetic acid (Ni-NTA) resin column. The SDS-PAGE analysis of the purified recombinant AaPGRP-LB (rAaPGRP-LB) protein revealed a predominant single band, indicative of a protein with an estimated molecular weight of 20 kDa (Figure 7).

### 2.6. In Vitro Verification of rAaPGRP-LB Function by Agglutination Assays

**Figure 8:**
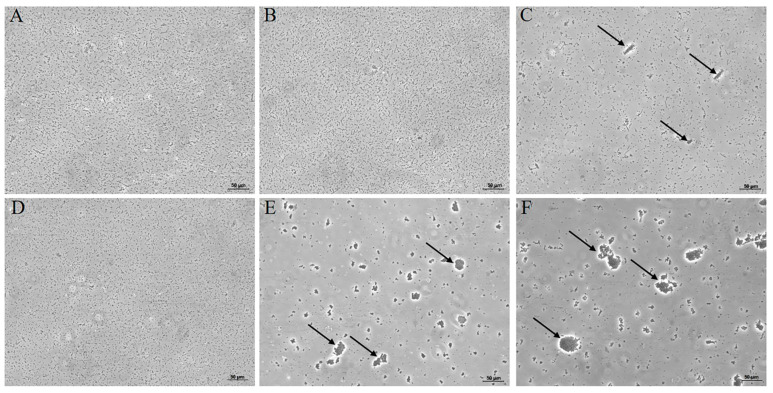
The agglutination effect of recombinant AaPGRP-LB (rAaPGRP-LB) protein. (**A**) *E. coli* + Tris; (**B**) *E. coli* + rAaPGRP-LB; (**C**) *E. coli* + rAaPGRP-LB + Zn^2+^; (**D**) *S. aureus* + Tris; (**E**) *S. aureus* + rAaPGRP-LB; (**F**) *S. aureus* + rAaPGRP-LB + Zn^2+^. The arrows indicate the sites where the rAaPGRP-LB protein and bacteria undergo agglutination.

*E. coli* and *S. aureus* were utilized to valuate the agglutination capability of rAaPGRP-LB. Without rAaPGRP-LB, no agglutination of *E. coli* or *S. aureus* was observed (Figure 8A,D). The introduction of rAaPGRP-LB to *E. coli* did not result in bacterial aggregation (Figure 8B). Conversely, a significant agglutination of *S. aureus* was observed after the introducton of rAaPGRP-LB (Figure 8E). Importantly, the addition of rAaPGRP-LB and Zn^2+^ to the *E. coli* system induced aggregation, in contrast to the group treated with rAaPGRP-LB alone (Figure 8C). Furthermore, the presence of rAaPGRP-LB increased in both the number and size of agglutination clusters in *S. aureus* (Figure 8F). These findings indicate that Zn^2^+ can enhance the bacterial agglutination ability of rAaPGRP-LB to a certain extent.

### 2.7. Inhibition Effects of rAaPGRP-LB on Bacterial Growth

We hypothesized that rAaPGRP-LB exhibits amidase activity, facilitating the hydrolysis of bacterial peptidoglycan and exhibiting antibacterial properties similar to other recombinant PGRP proteins. Considering the established Zn^2+^-dependence of PGRPs’ bactericidal activity [36], we examined the Zn^2+^ dependency of rAaPGRP-LB’s antibacterial effects. We evaluated the inhibitory effects of rAaPGRP-LB on the proliferation of *E. coli* (Table 1) and *S. aureus* (Table 2). In the absence of Zn^2+^, *E. coli* and *S. aureus* exhibited a similar lag phase but attained a lower maximum cell density than the control group. Conversely, in the presence of Zn^2+^, the growth curves of *E. coli* and *S. aureus* were significantly inhibited by rAaPGRP-LB. These results suggest that the antibacterial activity of rAaPGRP-LB is Zn^2+^-dependent and effectively inhibits the growth of *E. coli* and *S. aureus*.

## 3. Discussion

Insects possess an innate immune system capable of neutralizing microbial threats. PGRPs are essential in this defense because they recognize PGN on the surface of bacteria. Upon recognition, PGRPs activate the Toll and Imd signaling pathways, initiating a cascade of immune responses to combat infections [8,22]. *Ae. albopictus* is a primary vector for dengue fever transmission [37]. The recent extensive application of insecticides has led to the emergence of resistance in this species, posing a significant threat to public health [38]. Consequently, a comprehensive understanding of the immune defense mechanisms of *Ae. albopictus* is crucial for investigating new strategies in vector control and disease prevention.

Herein, we cloned the full-length cDNA sequence of *AaPGRP-LB*, conducted bioinformatics analyses, and performed functional studies. Functional domain analysis revealed that the AaPGRP-LB contains a signal peptide, indicating that PGRP-LB is probably a secreted protein that functions in the extracellular environment to recognize and bind bacterial peptidoglycan.

Studies have demonstrated that the analysis of conserved motifs and structural domains in proteins plays a critical role in elucidating their functional implications [39]. Most PGRPs demonstrating amidase activity contain the conserved HIS-TYR-HIS-THR-CYS motif [40]. Multiple sequence alignment confirmed that AaPGRP-LB retains the five essential amidase active sites of HIS-TYR-HIS-THR-CYS. Additionally, AaPGRP-LB contains a conserved PGRP/amidase domain homologous to the enzyme amidase. These findings are consistent with those of the reports in *D. melanogaster* that DmPGRP-LB possesses amidase activity [32,35,41]. Furthermore, phylogenetic analysis of AaPGRP-LB with PGRP proteins from various mosquitoes and insects demonstrated the accurate annotation of AaPGRP-LB as it clustered with orthologues, especially with DmPGRP-LB, supported by high bootstrap values.

We predicted the tertiary structure of AaPGRP-LB, which demonstrates a significant similarity to the tertiary structure of AaPGRP-LB and DmPGRP-LB, suggesting that the AaPGRP-LB protein exhibits a high degree of similar biological functions with DmPGRP-LB. The *DmPGRP-LB* gene encodes three distinct protein variants, two cytoplasmic (DmPGRP-LB^PA^ and DmPGRP-LB^PD^) and one secreted (DmPGRP-LB^PC^) [42]. DmPGRP-LB^PC^ cleaves PGN in the gut lumen, while the cytoplasmic variants degrade PGN within enterocytes [42]. Additional research in *D. melanogaster* demonstrated that the Y78F mutation in DmPGRP-LB inhibited PGN cleavage, while other mutations only reduced activity [43]. The molecular docking results demonstrate that AaPGRP-LB can effectively bind to the DAP-type and LYS-type PGNs, with a notable high binding affinity for TCT and MTP. The binding sites of AaPGRP-LB with TCT include TYR-92, while those with MTP comprise TYR-92 and CYS-174. The essential residues, TYR-92 and CYS-174, correspond to the amidase catalytic sites and are pivotal in the binding process. These findings indicate that AaPGRP-LB has a stronger effect against Gram-positive bacteria than against Gram-negative bacteria.

Following sequence identification and annotation, we assessed the response of *AaPGRP-LB* to bacterial stimuli through oral administration of the Gram-negative bacterium *E. coli* or the Gram-positive bacterium *S. aureus*. *AaPGRP-LB* was activated by various bacterial infections, indicating a general response to diverse bacterial stimuli. In *Ae. aegypti*, the *Ae. aegypti PGRP-LB* is similarly activated by both bacteria types [30]. *Ae. aegypti PGRP-LB* exhibits a more significant reaction to *E. coli*, while *Culex pipiens PGRP-LB* (*CpPGRP-LB*) exhibits an increased response to *S. aureus*. Similarly, the two *PGRP-LB* subtypes in *Anopheles gambiae*, *AgPGRP-LBa* and *AgPGRP-LBb*, are inducible by both bacteria, although with varying degrees of induction [44]. However, *DmPGRP-LB*, though untested against Gram-positive bacteria, was induced by a Gram-negative bacteria, *Erwinia carotovora* [40], indicating a consistent response with *Ae. albopictus*. The observed functional disparities among PGRPs may originate from multiple factors, including bacterial species variation, infection route differences, or intrinsic genetic variability among mosquito species. Previous investigations have established that blood feeding activates both Toll and Imd signaling pathways in the midgut of *Anopheles stephensi* [45]. Experimental studies employing bacterial injection in *Ae. aegypti* revealed critical roles for PGRPs in modulating vector immune responses [30]. Extending these findings, our current work employs sugar meals containing bacterial pathogens to demonstrate that AaPGRP-LB exerts essential regulatory functions in *Ae. albopictus*’ immune defense against both Gram-negative and Gram-positive bacterial challenges. In future research, the immune response mechanisms of AaPGRP-LB in *Ae. albopictus* will be investigated using blood meals or injections.

To investigate the immune function of AaPGRP-LB, we conducted agglutination assays to verify the function of rAaPGRP-LB. It revealed that rAaPGRP-LB exhibited a unique agglutination ability toward *E. coli* or *S. aureus* in the presence of Zn^2+^. This indicates that rAaPGRP-LB can serve as the receptor for recognizing these bacteria. Furthermore, rAaPGRP-LB exhibits significant antibacterial activity against *E. coli* and *S. aureus* in the presence of Zn^2+^, indicating that it possesses amidase activity, consistent with our previous predictions. The DmPGRP-SB1 is a protein exhibiting antibacterial activity conferred by its amidase activity [46]. Similarly, in *Rhynchophorus ferrugineus*, the recombinant RfPGRP-LB protein can significantly agglutinate *E. coli* and *S. aureus* in the presence of Zn^2+^. However, it exhibits antibacterial activity solely against *E. coli*, without significant antibacterial effect on *S. aureus* [36]. This variability indicates that the functionality of PGRP-LB proteins may differ among species. We hypothesized that AaPGRP-LB, as a secreted protein, is significantly up-regulated during foreign pathogens infection, synthesizing sufficient protein to recognize and bind the pathogens. In addition, the interaction of AaPGRP-LB with PGN was only evaluated bioinformatically. In further studies, experiments were performed using preparations of DAP-PGN and LYS-PGN and determine the amidase activity of AaPGRP-LB. Meanwhile, the role of AaPGRP-LB in the Toll or IMD immune signaling pathways and its function in activating antimicrobial peptide expression to eliminate bacteria warrant further investigation.

## 4. Materials and Methods

### 4.1. Mosquito Collection and Rearing

The mosquito specimens utilized in this study were *Ae. albopictus* of the Fuzhou strain, previously collected from the field and preserved in our laboratory [47]. The environmental conditions within the breeding chamber were carefully controlled, maintaining a temperature of 26 ± 2 °C, relative humidity of 70 ± 10%, and a photoperiod of 16 h of light followed by 8 h of darkness.

### 4.2. RNA Isolation and Amplification of AaPGRP-LB

Total RNA was extracted from *Ae. albopictus* specimens using the TRIpure Reagent (Cat No. RN0102; Aidlab, Beijing, China) according to the manufacturer’s instructions. The RNA concentration was measured using a NanoDrop One spectrophotometer (Thermo, Waltham, MA, USA), and the quality of the RNA was assessed by a 260/280 nm ratio between 1.8 and 2.1, indicating acceptable RNA quality. First-strand cDNA synthesis was performed using the NovoScript^®^Plus All-in-one 1st Strand cDNA Synthesis SuperMix (gDNA purge) kit (Cat No. E047-01B; Novoprotein, Beijing, China) according to the manufacturer’s instructions. This cDNA was employed as a template for the amplification of the *AaPGRP-LB* gene, using TransStart FastPfu DNA Polymerase (Cat No. AP221-11; TransGen, Beijing, China) with specific primers (F: 5′-ATGTACCTGAAGGGTGAAG-3′ and R: 5′-CTATGTTCTAATACTGTTGGG-3′). The PCR conditions were as follows: initial denaturation at 95 °C for 5 min, followed by 30 cycles of denaturation at 95 °C for 30 s, annealing at 55 °C for 30 s, and extension at 72 °C for 1 min, with a final extension at 72 °C for 10 min. The amplified product was purified and cloned into the pEASY-Blunt Cloning Kit (Cat No. CB101-01; TransGen, Beijing, China) and transformed into *E. coli* strain DH5α (Cat No. CB101-01; Tiangen, Beijing, China) for sequencing at Sangon Biotech (Shanghai, China).

### 4.3. Identification of AaPGRP-LB Gene

The DNA sequence was translated and the conserved domain of the AaPGRP-LB protein was analyzed using DNAMAN software (version 7.0) and NCBI CDD tools [48] (https://www.ncbi.nlm.nih.gov/Structure/cdd/wrpsb.cgi, accessed on 26 July 2024), respectively. The functional sites of the AaPGRP-LB protein was analyzed using the InterPro database (http://www.ebi.ac.uk/interpro/, accessed on 26 July 2024). The signal peptides were predicted utilizing the SignalP 5.0 server (https://services.healthtech.dtu.dk/services/SignalP-5.0/, accessed on 26 July 2024). The molecular weight (MW) and theoretical isoelectric point (pI) of deduced AaPGRP-LB protein was calculated using the ExPASy (Expert Protein Analyst System) platform online tool Compute pI/Mw (http://web.expasy.org/compute_pi/, accessed on 26 July 2024).

### 4.4. Sequence Alignment and Phylogenetic Analysis of AaPGRP-LB

The multiple sequence alignment of PGRP-LBs was performed using ClustalX (version 1.83) and visualized with GeneDoc software (version 2.7). A neighbor-joining (NJ) phylogenetic tree was performed using MEGA 7 software (version 7.0.26) based on the aligned PGRP-LBs protein sequences from different species (parameters selected: p-distance, pairwise deletion, 1000 bootstrap replicates).

### 4.5. Structure Prediction and Molecular Docking of AaPGRP-LB

The three-dimensional (3D) structure of AaPGRP-LB was predicted utilizing AlphaFold (version 2.3.2), while the 3D structure of DmPGRP-LB was obtained from the AlphaFold Protein Structure Database (https://alphafold.ebi.ac.uk/, accessed on 26 July 2024). Molecular structure superposition was performed using PyMOL software (version 3.0.0). The molecular docking protocol was implemented as follows: Autodock Vina (version 1.1.2) was employed as the docking algorithm; a grid box covering the entire protein was used to enable unbiased exploration of potential binding sites; the Vina scoring function evaluated ligand-protein binding affinities; 10 independent docking runs with an exhaustiveness value of 8 were performed per ligand–protein pair to ensure reproducibility. The optimal docking results were visualized with PyMOL software (version 3.0.0). TCT (PDB: 2F2L) and MTP (PDB: 1TWQ) serve as representations of DAP-type PGN and LYS-type PGN, respectively [49]. Structure files were sourced from the Protein Data Bank (PDB) database.

### 4.6. Expression Analysis of AaPGRP-LB by Reverse Transcription (RT)-qPCR

To investigate the potential roles of the *AaPGRP-LB* gene in the immune response of *Ae. albopictus*, we analyzed its transcriptional response after bacterial infections. *Ae. albopictus* was subjected to oral infection using Gram-positive bacteria *S. aureus* and Gram-negative bacteria *E. coli* DH5α. The *S. aureus* strains were provided by the Department of Pathogen Biology, School of Basic Medical Sciences, Fujian Medical University. The bacterial cultures were incubated overnight at 37 °C in Luria–Bertani (LB) medium, achieving an optical density (OD) of 0.6 at 600 nm (composition: 1000 mL distilled water, 10 g tryptone, 5 g yeast extract, 10 g NaCl, pH 7.2). Subsequently, 1 mL of each bacterial culture was centrifuged at 5000× *g* for 5 min at 4 °C. The supernatant was discarded, and the resulting cell pellet was washed thrice with 1 mL of sterilized phosphate-buffered saline (PBS; 137 mM NaCl, 2.7 mM KCl, 10 mM Na_2_HPO_4_, 2 mM K_2_HPO_4_, pH 7.4). The cell pellet was resuspended in 1 mL of a 10% sterilized glucose solution for experimental use.

In the pathogenic bacterial infection experiment, a cohort of healthy adult mosquitoes was systematically randomized into three experimental groups, the control group, the *E. coli* group, and the *S. aureus* group, each comprising 100 age-matched mosquitoes. For mosquito feeding, 1 mL of either the bacterial suspension or the 10% glucose solution was applied to the surface of sterilized medical gauze. Adult mosquitoes were allowed to feed continuously on the sucrose-bacteria mixture or the 10% glucose solution as a control. Surviving mosquitoes were randomly sampled (n = 20 per group) from each group for analysis at 6, 12, and 24 h after treatment. Each experimental group was performed in triplicate to ensure biological replication.

The procedures for RNA extraction and cDNA synthesis were executed as previously described. RT-qPCR was performed utilizing the NovoScript^®^ SYBR qPCR SuperMix Plus kit (Cat No. E096-01A; Novoprotein, Beijing, China) with a 20 μL reaction volume containing 2 μL cDNA and 125 nM of each primer. The *AaPGRP-LB* fragment was amplified with primers *AaPGRP-LB*F (5′-ACTTCAGCGTGTTTGCCCTAC-3′) and *AaPGRP-LB*R (5′-TTGAATCCTCGTCCCTCGTAG-3′). The *AaRPS7* gene was amplified as an internal control with primers *AaRPS7*F (5′- TGGTCCGTGAGTTGGAGAAGA-3′) and *AaRPS7*R (5′- GTGGTCTGCTGGTTCTTGTCC-3′). The thermal cycling protocol comprised an initial denaturation at 95 °C for 10 min, followed by 40 cycles of 95 °C for 15 s, and 60 °C for 1 min, with a dissociation step. Gene expression levels were normalized to *AaRPS7* using the 2^−∆∆Ct^ method.

### 4.7. Expression and Purification of Recombinant AaPGRP-LB Protein of Ae. albopictus

The cDNA encoding the predicted AaPGRP-LB protein, excluding its signal peptide, was cloned into the expression vector pET-28a by Beijing Tsingke Biotech Co., Ltd., Beijing, China. The recombinant plasmid, labeled pET-28a-His-AaPGRP-LB, was confirmed through sequencing and subsequently introduced into *E. coli* BL21(DE3) (Cat No. CB105-02; Tiangen, Beijing, China). The modified *E. coli* was cultured in LB medium supplemented with kanamycin at 37 °C. When the OD_600_ reached 0.4–0.6, IPTG was introduced to the culture medium to achieve a final concentration of 0.6 mM. The culture was subsequently incubated at 16 °C with agitation at 150 rpm for 20 h.

Following cultivation, bacterial cells were harvested by centrifugation at 8000× *g* for 5 min at 4 °C. Cell lysis was performed using a Tris buffer (20 mM Tris-HCl, 0.10 mM NaCl, pH 8.0) combined with ultrasonication (90 W output power) employing intermittent cycles of 3 s pulses followed by 3 s intervals for a total duration of 30 min. The lysate was clarified by centrifugation at 12,000× *g* for 10 min at 4 °C, and the resulting supernatant was subjected to further purification. Recombinant proteins were isolated from the supernatant using HisTrap™ affinity chromatography columns (GE Healthcare, Chicago, IL, USA) according to the manufacturer’s protocol. Eluted fractions protein samples were mixed with 2 × loading buffer at a 1:1 ratio (20 μL total volume per lane) and resolved on 12% SDS-PAGE gels. Electrophoresis was conducted using a two-stage protocol: initial separation at 80 V for 30 min followed by 120 V for 90 min. Protein bands were visualized using Coomassie brilliant blue R-250 staining (Cat No. P0017B; Beyotime, Shanghai, China) and gel imaging was performed using the Tanon MINI Space 2000 gel imaging system (Tanon, Shanghai, China). Subsequently, eluted fractions containing the target protein were pooled and further purified by size-exclusion chromatography on a Superdex™ 200 Increase column (GE Healthcare, Chicago, IL, USA) using an AKTA pure protein purification system (GE Healthcare, Chicago, IL, USA). The purified protein was concentrated utilizing Amicon Ultra Centrifugal Filter Devices (Millipore, Burlington, MA, USA), and its concentration was quantified using a NanoDrop One spectrophotometer (Thermo, Waltham, MA, USA). The protein was stored at −80 °C.

### 4.8. Agglutination Assay of rAaPGRP-LB

The agglutination activity of rAaPGRP-LB against *E. coli* DH5α and *S. aureus* was evaluated to determine its role in bacterial agglutination. The bacteria were cultured separately in an LB liquid medium at 37 °C until the logarithmic phase, achieving an OD_600_ of approximately 0.6. The cultures were subsequently harvested and suspended in Tris buffer at 1 × 10^5^ CFU/mL. On a sterile glass slide, 10 μL of the bacteria suspensions were mixed with 25 μL rAaPGRP-LB (100 μg/mL) [50,51] or Tris buffer, with or without the addition of 1 μL ZnCl_2_ (10 mM). The mixtures were incubated at room temperature for 1 h and observed under Leica DM500 (Leica, Wetzlar, Germany).

### 4.9. Antibacterial Activity Assay of rAaPGRP-LB

The antibacterial activity of rAaPGRP-LB against *E. coli* DH5α and *S. aureus* was evaluated. The bacteria were cultured in an LB liquid medium at 37 °C until the logarithmic phase, indicated by an OD_600_ of approximately 0.6. The cultures were diluted 1:100 in fresh LB medium, and 100 μL of the *E. coli* DH5α and *S. aureus* dilutions were added into 96-well plates (200 μL/per well). Subsequently, 100 μL of the rAaPGRP-LB (100 μg/mL) was mixed with the bacteria, with or without 10 mM ZnCl_2_, utilizing Tris buffer as a control. The plates were incubated at room temperature with shaking at 200 rpm, and the OD_600_ was measured hourly for 12 h using the Multiskan FC microplate reader (Thermo, Waltham, MA, USA). Each experimental condition was performed in triplicate.

### 4.10. Statistical Analysis

Statistical Package for the Social Sciences software (version 25.0) was utilized for statistical analysis for the bacterial challenge experiments and rAaPGRP-LB antibacterial assays. Data visualization was performed using GraphPad Prism software (version 8.0.2). The results are expressed as means ± standard deviation (SD) derived from three biological replicates. An unpaired Student’s *t*-test was utilized to assess differences between two groups under normal data distribution, with Bonferroni correction applied when variance was homoscedastic, or Games–Howell correction when heteroscedasticity was detected. A repeated measures analysis of variance (ANOVA) was utilized for comparisons among three or more groups with repeated measures, followed by Bonferroni correction for homoscedastic variance or Games–Howell correction for heteroscedastic variance. A *p* < 0.05 was considered statistically significant.

## 5. Conclusions

This study demonstrated the indispensable role of AaPGRP-LB in the immune response of *Ae. albopictus*. Bioinformatics analysis, functional assays, and comparative studies revealed that AaPGRP-LB is a secretory amidase active protein that can recognize and degrade bacterial PGN, actively participating in the mosquito’s defense mechanisms. These insights pave the way for future research on leveraging mosquito immune pathways for innovative vector control and disease prevention strategies.

## Figures and Tables

**Table 1:** Data on the effect of rAaPGRP-LB on the growth inhibition of *Escherichia coli*.

Time (h)	Tris + *E. coli* (OD)	Tris + Zn^2+^ + *E. coli* (OD)	rAaPGRP-LB + *E. coli* (OD)	rAaPGRP-LB + Zn^2+^ + *E. coli* (OD)
0	0.107 ± 0.002	0.107 ± 0.001	0.113 ± 0.001 *	0.112 ± 0.002 #
1	0.107 ± 0.002	0.107 ±0.001	0.114 ± 0.001 *	0.111 ± 0.002 #
2	0.105 ± 0.001	0.108 ± 0.001	0.114 ± 0.001 *	0.112 ± 0.001 #
3	0.106 ± 0.002	0.109 ± 0.001	0.114 ± 0.001 *	0.112 ± 0.001 #
4	0.112 ± 0.003	0.115 ± 0.001	0.117 ± 0.001 *	0.113 ± 0.001
5	0.115 ± 0.002	0.117 ± 0.001	0.120 ± 0.002 *	0.118 ± 0.002
6	0.124 ± 0.002	0.124 ± 0.001	0.121 ± 0.002	0.125 ± 0.002
7	0.137 ± 0.003	0.136 ± 0.002	0.123 ± 0.002 *	0.140 ± 0.002
8	0.154 ± 0.003	0.155 ± 0.003	0.134 ± 0.004 *	0.145 ± 0.001 #
9	0.188 ± 0.001	0.186 ± 0.003	0.166 ± 0.007 *	0.147 ± 0.001 #
10	0.227 ± 0.002	0.225 ± 0.003	0.197 ± 0.002 *	0.148 ± 0.001 #
11	0.282 ± 0.002	0.281 ± 0.001	0.239 ± 0.002 *	0.149 ± 0.001 #
12	0.314 ± 0.002	0.313 ± 0.002	0.267 ± 0.002 *	0.150 ± 0.001 #

* *p* < 0.05, Tris + *E. coli* vs. rAaPGRP-LB + *E. coli*; # *p* < 0.05, Tris + *E. coli* vs. rAaPGRP-LB + Zn^2+^ + *E. coli*.

**Table 2:** Data on the effect of rAaPGRP-LB on the growth inhibition of *Staphylococcus aureus*.

Time (h)	Tris + *S. aureus* (OD)	Tris + Zn^2+^ + *S. aureus* (OD)	rAaPGRP-LB + *S. aureus* (OD)	rAaPGRP-LB + Zn^2+^ + *S. Aureus* (OD)
0	0.107 ± 0.003	0.106 ± 0.002	0.114 ± 0.002 *	0.113 ± 0.001 #
1	0.107 ± 0.002	0.107 ± 0.002	0.114 ± 0.002 *	0.114 ± 0.001 #
2	0.111 ± 0.003	0.110 ± 0.002	0.116 ± 0.002	0.129 ± 0.002 #
3	0.118 ± 0.004	0.116 ± 0.002	0.122 ± 0.002	0.135 ± 0.002 #
4	0.138 ± 0.002	0.139 ± 0.002	0.141 ± 0.001 *	0.137 ± 0.001
5	0.158 ± 0.003	0.156 ± 0.002	0.152 ± 0.002 *	0.140 ± 0.001 #
6	0.211 ± 0.005	0.209 ± 0.004	0.184 ± 0.002 *	0.143 ± 0.002 #
7	0.302 ± 0.003	0.296 ± 0.001	0.242 ± 0.001 *	0.144 ± 0.002 #
8	0.375 ± 0.004	0.372 ± 0.001	0.291 ± 0.003 *	0.145 ± 0.002 #
9	0.443 ± 0.002	0.442 ± 0.001	0.352 ± 0.005 *	0.146 ± 0.002 #
10	0.492 ± 0.006	0.493 ± 0.003	0.408 ± 0.005 *	0.147 ± 0.002 #
11	0.552 ± 0.003	0.553 ± 0.002	0.455 ± 0.002 *	0.146 ± 0.001 #
12	0.616 ± 0.002	0.617 ± 0.002	0.483 ± 0.002 *	0.146 ± 0.001 #

* *p* < 0.05, Tris + *S. aureus* vs. rAaPGRP-LB + *S. aureus*; # *p* < 0.05, Tris + *S. aureus* vs. rAaPGRP-LB + Zn^2+^ + *S. aureus*.

## Data Availability

All data generated and analyzed during this study are included in this article. Further inquiries can be directed to the corresponding author.

## Abbreviations

3D	three-dimensional
AMPs	antimicrobial peptides
ANOVA	analysis of variance
CDD	conserved domain database
IPTG	isopropyl β-D-1-thiogalactopyranoside
MTP	muramyl tripeptide
Ni-NTA	nickel-nitrilotriacetic acid
OD	optical density
ORF	open reading frame
PBS	phosphate-buffered saline
PGRPs	peptidoglycan recognition proteins
PGNs	peptidoglycans
PPO	prophenoloxidase
PRRs	pattern recognition receptors
RT-qPCR	real-time quantitative polymerase chain reaction
SD	standard deviation
TCT	tracheal cytotoxin
